# Traffic jam in lung capillaries: inter-organ communication impedes gas exchange after acute kidney injury

**DOI:** 10.1172/JCI192917

**Published:** 2025-05-15

**Authors:** Ulrich Matt, Susanne Herold

**Affiliations:** 1 Department of Medicine V, Internal Medicine, Infectious Diseases and Infection Control, University Hospital Giessen and Marburg (UKGM), member of the German Center for Infection Research (DZIF), member of the German Center for Lung Research (DZL) and the Institute for Lung Health (ILH), Giessen, Germany.; 2 Excellence Cluster Cardiopulmonary Institute (CPI), Giessen, Germany.

## Abstract

Acute kidney injury (AKI) is a frequent complication in critically ill patients and triggers a systemic inflammatory response that can contribute to lung injury, ultimately worsening clinical outcomes. However, diagnostic and therapeutic strategies remain unavailable. In this issue of the *JCI*, Komaru et al. explored leukocyte trafficking and vascular pooling following AKI in mice as an underlying mechanism of acute lung injury. Using intravital microscopy, the authors observed rapid accumulation of neutrophils in pulmonary capillaries within minutes of AKI onset. These neutrophils followed monocytes and slowed blood flow. Notably, disruption of this process improved oxygenation. The findings provide insights into this complex inter-organ crosstalk and open avenues for future research.

## How kidneys affect respiratory function

Inter-organ crosstalk relies on complex pathophysiological communication loops between different organs in the human body that are crucial for maintaining homeostasis and resilience to tissue injury. Dysregulated inter-organ crosstalk, however, can exacerbate disease trajectories and negatively affect clinical outcomes. Examples are the increased incidence of cardiovascular events following pneumonia (1), or the clinically well-described cardio-renal syndrome (2), among others (3–5). Some of these interactions are confined to systemic, trans-organ activation of circulating and local immune cells (3, 4, 6), but the underlying mechanisms are certainly manifold. Understanding these interactions is essential for developing targeted therapies and improving patient outcomes.

Crosstalk between kidneys and lungs is a frequent and clinically relevant, albeit widely unexplained, observation. The majority of available data highlight the effect of acute lung injury (ALI) and acute respiratory distress syndrome (ARDS) on renal function (7). Acute kidney injury (AKI) results in volume overload due to oliguria or anuria, leading to edema. In addition, impaired oxygenation in the absence of volume overload and unchanged pulmonary artery wedge pressure have been described (8, 9). Autopsy studies revealed proteinaceous edema formation, hyaline membranes, and leukocyte infiltration in the lungs after AKI, which led investigators to suggest the term “uremic pneumonitis” (10). Clinically, AKI-induced hypoxemic lung injury worsens outcome but is often not recognized as a unique pathomechanistic entity, as it often coexists with ARDS and/or fluid overload (11). AKI triggers a systemic inflammatory response that involves an increase of circulating neutrophils and monocytes (11). Notably, compared with end-stage renal failure, AKI has a worse outcome, signifying acute pathophysiological alterations that worsen organ functions (12). Importantly, mechanical ventilation due to respiratory failure was found as the only factor that increased mortality in multivariate analyses, highlighting the link between AKI and ALI (12).

## Classical monocytes halt neutrophils within the lung vasculature

**Figure 1:**
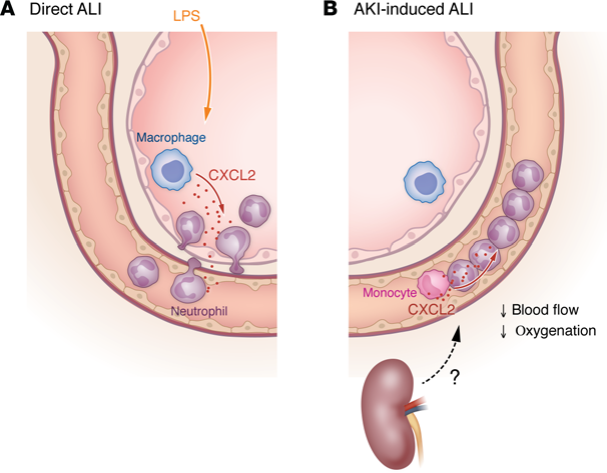
Direct and AKI-induced ALI result in different neutrophil chemotaxis patterns. (**A**) Induction of direct ALI via LPS administration elicits a proinflammatory response from alveolar macrophages, leading to neutrophil infiltration into the bronchoalveolar space. (**B**) In contrast, induced AKI triggers a chemotactic response via classical monocytes, resulting in the formation of trains of less deformable neutrophils within the pulmonary vasculature. These trains decrease blood flow and impair oxygenation.

In this issue of the *JCI,* Komaru et al. used elegant intravital 2-photon microscopy analysis in a murine model of bilateral renal perfusion injury (13). As early as five to ten minutes after AKI, neutrophils and monocytes emerged in the lung vasculature. Strikingly, the leukocytes migrated to the lung without exiting the blood vessels (Figure 1). Slowly moving CCR2^+^ monocytes appeared to induce trains of neutrophils within the capillaries, resulting in reduced blood flow. While more than 99.5% of neutrophils remained within the lung vasculature, intrapulmonary LPS administration resulted in the extravasation of approximately 80% of neutrophils into the bronchoalveolar space. Hypoxemia was detectable two to six hours after ischemic kidney injury, and depletion of neutrophils led to the restoration of blood flow and oxygenation, suggesting that reduced flow by neutrophil capillary pooling impaired oxygenation. Intravital imaging revealed a more rounded shape of neutrophils, along with stronger F-actin signal after AKI compared with sham treatment. Thus, the authors suggest that reduced neutrophil stiffness contributed to impaired extravasation (13). However, these neutrophils were capable of reaching the bronchoalveolar space when a neutrophil chemoattractant, CXCL2, was instilled intratracheally. Ultimately, in contrast to alveolar macrophages and nonclassical monocytes, depletion of classical monocytes abrogated neutrophil accumulation in lung capillaries. Cell communication analysis of single-cell RNA-Seq data pointed toward CXCL2-based, compartmentalized communication between classical monocytes and neutrophils within the capillary lumen. Of note, resident alveolar macrophages revealed no proinflammatory reprogramming after AKI as compared with direct lung injury and, in particular, no expression of CXCL2, adding to the above-mentioned mechanisms of vascular retainment of neutrophils. Additionally, AKI triggered microthrombosis, as evidenced by antiplatelet and antifibrinogen staining. Together, Komaru and authors discovered a neutrophil-dependent mechanism explaining impaired oxygenation in the absence of fluid overload after AKI that may provide new avenues for treatment (13).

## Conclusions and outlook

Komaru and colleagues speculated that the neutrophil retention signal in the pulmonary vasculature may be elicited as a counter-mechanism to prevent an overt systemic inflammatory response caused by AKI (13). While they proposed to intervene with neutrophil trains as a therapeutic approach, a causal approach might be to counteract the signals that lead to such intravascular chemotactic events in the lung after AKI.

Preclinical studies have identified cytokines and other immunoregulatory molecules as mediators of kidney-lung crosstalk (14). Since experimental nephrectomy does not prevent lung injury, it indicates that the kidneys are not the source of inflammatory mediators and suggests that uremic factors may be responsible for triggering their release (15). However, since leukocytes appear within minutes, uremic factors are probably not responsible for this immediate response. Previous research has shown that IL-6 promotes neutrophil accumulation in the lungs through pulmonary CXCL1 expression. Yet, as IL-6 injection alone does not trigger CXCL1 expression, additional unidentified factors must be involved in neutrophil chemotaxis (16). Previous studies from the Herrlich laboratory identified osteopontin as a key factor released from the kidneys following AKI, triggering ALI (17). Using the same AKI model, the authors found that osteopontin released from kidney tubule cells induced lung endothelial leakage, inflammation, and respiratory failure (17). It remains unclear whether osteopontin or other known mediators such as IL-6 stimulate CXCL2 release by classical monocytes in the lung vasculature, a question that warrants further investigation.

Both human and preclinical studies applying ischemia-reperfusion injury regularly describe lung edema formation and endothelial barrier disruption following AKI, likely contributing to impaired oxygenation (17–20). In the present study, Komaru and authors did not confirm edema formation by histopathological assessment, and alveolar edema assessment via bronchoalveolar lavage protein measurement was not presented (13). Thus, the relative contributions of neutrophil trains and edema formation remain uncertain, as does their respective effect on oxygenation impairment under experimental hyperoxia induced by mechanical ventilation with 100% O_2_. Future research should also explore the contribution of microthrombosis to gas exchange dysfunction in this process. Linking these findings could provide a more comprehensive understanding of AKI-associated hypoxemia and the direct and indirect contribution of barrier dysfunction. Investigation of the compartment-specific functions of factors such as CXCL2 and osteopontin could identify potential therapeutic targets to mitigate AKI-induced respiratory failure.

## Version 1 05/15/2025

Electronic publication
